# Zinc Status of Vegetarians during Pregnancy: A Systematic Review of Observational Studies and Meta-Analysis of Zinc Intake

**DOI:** 10.3390/nu7064512

**Published:** 2015-06-05

**Authors:** Meika Foster, Ursula Nirmala Herulah, Ashlini Prasad, Peter Petocz, Samir Samman

**Affiliations:** 1 Department of Human Nutrition, University of Otago, PO Box 56, Dunedin 9054, New Zealand; E-Mail: meika.foster@otago.ac.nz; 2 Discipline of Nutrition and Metabolism, School of Molecular Bioscience, University of Sydney, NSW 2006, Australia; E-Mail: uher9297@uni.sydney.edu.au; 3 Department of Statistics, Macquarie University, NSW 2109, Australia; E-Mails: ashlini-ashika.prasad@students.mq.edu.au (A.P.); peter.petocz@mq.edu.au (P.P.)

**Keywords:** zinc, vegetarian, pregnancy, intake, biomarker, status, requirement, diet

## Abstract

Pregnant women are vulnerable to a low zinc status due to the additional zinc demands associated with pregnancy and foetal development. The present systematic review explores the relationship between habitual vegetarian diets and dietary zinc intake/status during pregnancy. The association between vegetarian diets and functional pregnancy outcome also is considered. A literature search was conducted of MEDLINE; PubMed; Embase; the Cochrane Library; Web of Science; and Scopus electronic databases up to September 2014. Six English-language observational studies qualified for inclusion in the systematic review. A meta-analysis was conducted that compared the dietary zinc intake of pregnant vegetarian and non-vegetarian (NV) groups; the zinc intake of vegetarians was found to be lower than that of NV (−1.38 ± 0.35 mg/day; *p* < 0.001); and the exclusion of low meat eaters from the analysis revealed a greater difference (−1.53 ± 0.44 mg/day; *p* = 0.001). Neither vegetarian nor NV groups met the recommended dietary allowance (RDA) for zinc. In a qualitative synthesis; no differences were found between groups in serum/plasma zinc or in functional outcomes associated with pregnancy. In conclusion; pregnant vegetarian women have lower zinc intakes than NV control populations and both groups consume lower than recommended amounts. Further information is needed to determine whether physiologic adaptations in zinc metabolism are sufficient to meet maternal and foetal requirements during pregnancy on a low zinc diet.

## 1. Introduction

The involvement of zinc in numerous biological processes, including enzyme action, regulation of gene expression, and cell signalling, underscores the importance of this essential trace element in health [1,2]. Severe zinc deficiency is characterised by impaired growth, delayed sexual and bone maturation, impaired immunity, and diarrhoea. Although severe zinc deficiency is rare in affluent countries, less acute deficiency states resulting from an inadequate intake of bioavailable zinc are believed to be highly prevalent [3].

Zinc is widely distributed in foods, with meat, fish, shellfish, and poultry being the major contributors of bioavailable zinc in the adult omnivorous diet [4]. In contrast, zinc is less bioavailable and likely to be present in lower amounts when obtained from plant-derived compared to animal food sources [5], suggesting that careful planning is required to ensure adequate amounts of absorbable zinc are present in a vegetarian diet.

In a recent meta-analysis [6], dietary zinc intakes were found to be lower in adults who follow habitual vegetarian diets compared to omnivorous control groups. Although serum zinc concentrations also were lower in vegetarians, no adverse health consequences attributable to the lower dietary zinc intake were apparent, presumably because of homeostatic mechanisms that allow healthy adults to adapt to a vegetarian diet [7]. The difference in intake may be important, however, for vegetarians with additional zinc requirements, including those who are pregnant. Pregnant women are vulnerable to a low zinc status due to the additional zinc demands associated with pregnancy and foetal growth and development, with late pregnancy being the period of greatest need [8].

The aim of the present paper is to undertake a systematic review and, where appropriate, meta-analysis of observational studies that investigate measures of zinc intake and status during pregnancy in women following habitual vegetarian diets compared to their omnivorous counterparts. Where possible, the relationship between zinc intake/status and functional pregnancy outcome in these populations also will be described.

## 2. Methods

### 2.1. Search Strategy

**Figure 1:**
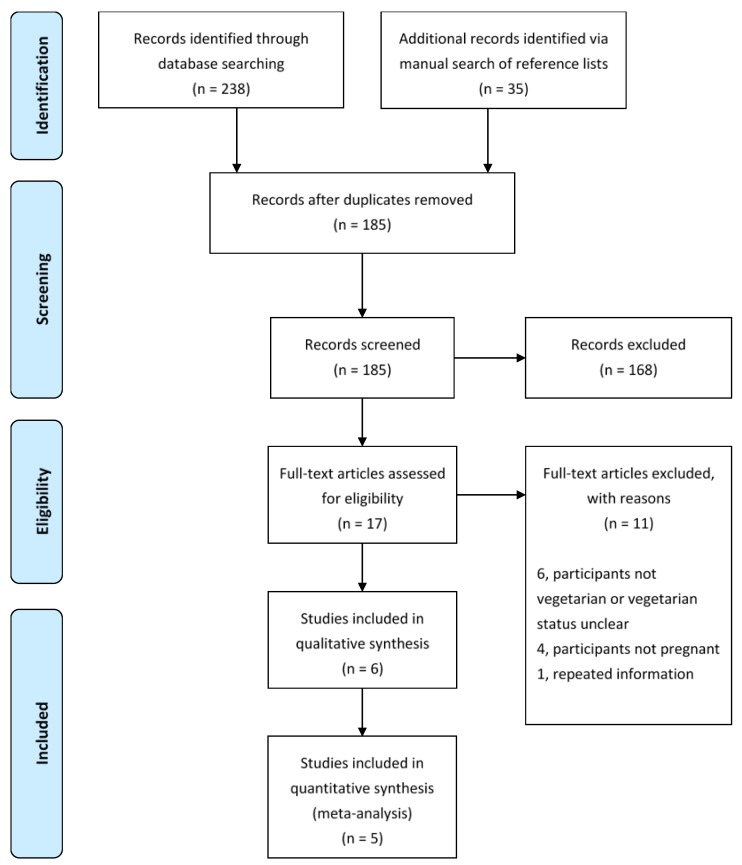
Flowchart detailing identification and selection of studies for inclusion in the review [9].

A literature search was conducted of MEDLINE, PubMed, Embase, the Cochrane Library, Web of Science, and Scopus electronic databases from the beginning of coverage to September 2014 using the keyword search strategy (‘zinc’ OR ‘Zn’) AND (‘pregnant’ OR ‘pregnanc*’) AND (‘plant-based’ OR ‘vegetarian*’ OR ‘vegan*’). Journal articles were restricted to human investigations published in English. Reference lists of retrieved studies were inspected for additional relevant articles. The PRISMA flowchart [9] describing the studies identified from the search strategy is depicted in Figure 1.

### 2.2. Study Selection

Observational studies published in English in peer-reviewed journals that examined zinc intake and/or status in vegetarian compared to non-vegetarian pregnant women fulfilled the criteria for inclusion in the systematic review. The title, abstract, and descriptors of each study identified in the literature search were screened to identify those that appeared eligible for full review. Three investigators (MF, SS, UH) independently reviewed each full report to determine if the depicted study did, in fact, meet the inclusion criteria. Studies excluded from further analysis [10,11,12,13,14,15,16,17,18,19,20], and the reasons for their exclusion, are provided in Table 1.

**Table 1:** Reasons for study exclusion.

Reason for Exclusion	Study Details
Participants not described as vegetarian; and no comparison group (6 studies)	Abebe *et al.*, 2008 [10]
	Fitzgerald *et al.*, 1993 [11]
	Huddle *et al.*, 1998 [12]
	Nguyen *et al.*, 2013 [13]
	Pathak *et al.*, 2004 [14]
	Pathak *et al.*, 2008 [15]
Participants not pregnant (4 studies)	Ellis *et al.*, 1987 [16]
	Grieger *et al.*, 2014 [17]
	Siyame *et al.*, 2013 [18]
	Waldmann *et al.*, 2003 [19]
Repeated information (1 study)	Abraham, 1982 [20] (preliminary data published in full in Abraham *et al.*, 1985 [21])

### 2.3. Data Extraction and Meta-Analysis

Data from all selected studies [21,22,23,24,25,26] were abstracted independently using an excel spreadsheet of common format by two investigators (MF and UH), followed by discussion with a third investigator (SS) to confirm the data. Study characteristics, including diet group definitions, sample size, country in which the study was conducted, and zinc status outcomes, are described in Table 2.

Diet groups were defined in accordance with our previous report in healthy adult vegetarians [6], as follows: lacto-vegetarian (V-L), ovo-lacto vegetarian (V-OL), vegetarian undefined (VU), low meat group (LoM), and non-vegetarian (NV). Vegetarian populations were reclassified as LoM for the purposes of the present meta-analysis in instances where the study definition of a vegetarian diet included limited amounts of meat, fish, or poultry. Mean outcome data were extracted, where available, for dietary zinc intake, concentrations of zinc in serum/plasma, urine, and hair, period of gestation, and birthweight of infants. Units were converted to International System of Units (SI) measures, where applicable, and measures of variability were recorded as standard error of the mean (SE). Outcomes with six or more lines of available data were deemed sufficient for meta-analysis to be conducted. Dietary zinc intake met the required threshold and a description of the data that was included in the meta-analysis of zinc intake is provided in Table 2; where data were available for more than one NV population, the group most similar in characteristics to the vegetarian population was used as the control group.

### 2.4. Quality Assessment

Two reviewers (MF and SS) independently assessed the methodological quality of each included study using the American Dietetic Association (ADA) Quality Criteria Checklist for Primary Research (modified for observational studies) [27]. Studies were assigned a quality rating of positive (*i.e.*, low risk of bias), neutral, or negative (*i.e.*, high risk of bias). Inter-investigator discrepancies in ratings of individual studies were resolved by consensus.

**Table 2:** Description of included studies.

Study (author, year)	Diet Group ^1^	*n* ^2^	Country	Biomarkers of Zn Status [Stage of Pregnancy when Measured ^3^]	Dietary Methodology for Zn Intake Measurement	Included in Meta-Analysis of Zn Intake? Yes/no; with Comments
King, Stein & Doyle, 1981 [22]	V-OL NV	9 6	USA	intake [trimester 3]	3 day estimated diet record	Yes; pregnant vegetarians compared to pregnant NV; non-pregnant vegetarian data not included
				plasma [trimester 3]		
				urine [trimester 3]		
				hair [trimester 3]		
Abu-Assal & Craig, 1984 [23]	LoM (consume meat < twice per month)	12	USA	intake [≥32]	3 day estimated diet record	Yes; dietary Zn data used; data including supplemental Zn not included
	NV (consume meat ≥ 4 times per week)	17		plasma [37 ± 2 ^4^] PP ^1^ plasma [11 ± 7 ^4^]		
Abraham *et al.*, 1985 [21]	V-L	134	UK	intake [trimester 1 (30%) ^5^]	7 day dietary recall	Yes; Hindu Asian vegetarian groups compared to NV counterparts; Muslim NV and European NV data not included
	V-OL	271				
	LoM (consume meat once every 2–4 weeks)	45				
	NV (‘regularly’ consume meat/fish/eggs/cheese)	225				
Campbell-Brown *et al.*, 1985 [24]	VU	57	UK	intake [1st antenatal visit]	7 day dietary recall	No; participants are sub-group of Abraham *et al.* [21], 1985 study
	NV (consume meat ≥ once per fortnight)	31		serum [booking, 20, 28, 36]		
				urine [booking, 20, 36]		
				hair [booking, 36]		
Ward *et al.*, 1988 [25]	VU	53	India	intake [28]	7 day dietary recall	Yes; Gujerat vegetarians compared to NV counterparts; historical data from Campbell-Brown *et al.*, 1985 [24] re Asian vegetarians and NV not included
	NV	20		plasma [28]		
Drake, Reddy & Davies, 1998 [26]	V-OL	31	UK	intake [V-OL: 25.0 ± 9.6 ^4^; NV: 24.3 ± 8.2 ^4^]	3 day estimated diet record	Yes; dietary Zn data used; data including supplemental Zn not included
	NV	69				

^1^ Abbreviations: LoM, low meat; NV, non-vegetarian; PP, postpartum; V-L, lacto-vegetarian; V-OL, ovo-lacto vegetarian; VU, vegetarian undefined; ^2^ *n* for Zn intake (values may differ for other outcome measurements); ^3^ expressed as trimester 1, 2, 3 or weeks of gestational age, unless otherwise stated; ^4^ mean ± SD; ^5^ further information not provided.

### 2.5. Statistical Analysis

Meta-analyses of dietary zinc intake (mg/day) were carried out using the Comprehensive Meta-Analysis package, version 2 (Biostat, 2005, Englewood, NJ, USA, www.meta-analysis.com). Results were generated using vegetarian (V-L, V-OL, VU or LoM) minus control group (NV) values and are summarised in the form of a forest plot. A second forest plot was generated that excluded LoM data to assess the effect on zinc intake of diets that adhere to strict interpretations of vegetarian dietary patterns. The random-effects model was utilised in each case rather than the fixed-effects approach as differences in study design among included studies precluded the assumption of a common effect size. Results are expressed as mean difference ± SE, with the standard error of difference calculated using the independence of vegetarian and control groups. Sensitivity analyses were performed to determine the impact of individual studies on effect sizes. Funnel plots of SE by mean were generated to assess publication bias. Statistical heterogeneity was assessed using a chi-squared statistic and calculation of *I*-squared. A *p*-value < 0.01 was interpreted as statistically significant.

## 3. Results

Six English-language, observational studies that investigated measures of zinc intake/status in vegetarian compared to non-vegetarian pregnant women [21,22,23,24,25,26] qualified for inclusion in the systematic review and are described in Table 2. Of these, five studies assessed functional outcome in pregnancy. The included studies were conducted in the United Kingdom (3 studies [21,24,26]), the United States of America (2 studies [22,23]) and India (1 study [25]), predominantly in the 1980s. Excluding the largest study (*n* = 675 [21]), the average number of subjects per study was 61.

### 3.1. Dietary Zinc Intake in Pregnancy

All six of the included studies compared the zinc intake of pregnant vegetarians and their respective omnivorous control groups, allowing meta-analyses to be conducted. Three of the studies [21,24,25] were generated by the same research group; one such study [24] was excluded from the meta-analysis as it was conducted in a sub-group of participants from the largest of the three collaborative studies [21]. Of the remaining 5 studies, zinc intake was measured in populations defined as V-OL (3 studies [21,22,26]), V-L (1 study [21]), VU (1 study [25]), and LoM (2 studies [21,23]).

**Figure 2:**
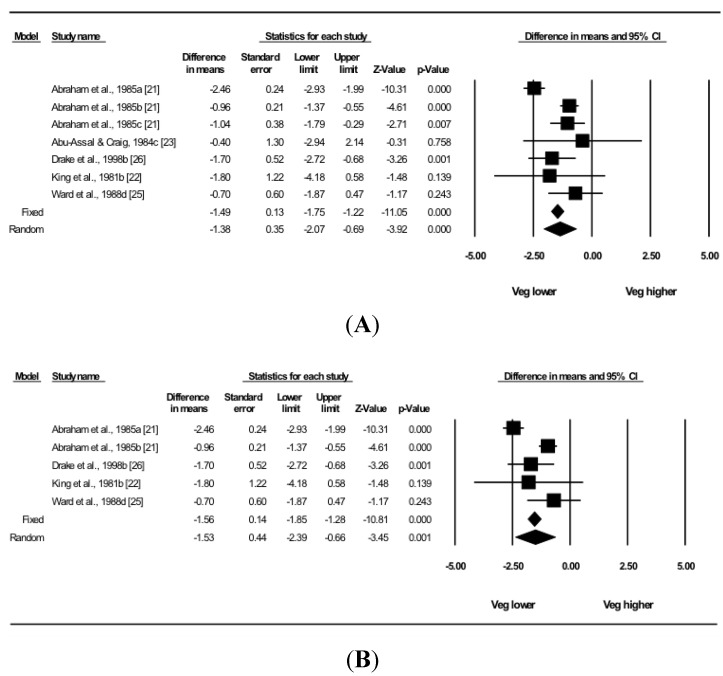
Analysis of dietary zinc intake (mg/day) compared to NV controls in (**A**) pregnant vegetarians; and (**B**) pregnant vegetarians with data from LoM excluded. Results are expressed as mean difference ± SE, with the standard error of difference calculated using the independence of vegetarian and control groups. a, lacto-vegetarian (V-L); b, ovo-lacto-vegetarian (V-OL); c, low meat (LoM); d, vegetarian undefined (VU).

In a meta-analysis of the five qualifying studies that compared the dietary zinc intake of pregnant vegetarian groups with NV controls, the zinc intake of pregnant vegetarians was found to be lower than that of NV (−1.38 ± 0.35 mg/day, *p* < 0.001; Figure 2A). Insufficient data were available to allow secondary analyses by vegetarian dietary pattern to be conducted; however, a second analysis was carried out that excluded the LoM data to assess the effect on zinc intake of diets that adhere to strict definitions of vegetarian dietary patterns. The results showed a lower zinc intake in pregnant vegetarians compared to NV, with a larger difference between the two groups in the amount consumed per day (−1.53 ± 0.44 mg/day, *p* = 0.001; Figure 2B).

In sensitivity analyses, removal in turn of each individual comparison resulted in no appreciable change to the results (all *p* ≤ 0.001). The heterogeneity test (Q = 27.0 on 6 df, *p* < 0.001) supported the use of the random-effects model, and the *I*-squared statistic (*I*^2^ = 77.8) quantified the large variability between studies. There was no evidence of publication bias in funnel plots of SE by mean.

**Figure 3:**
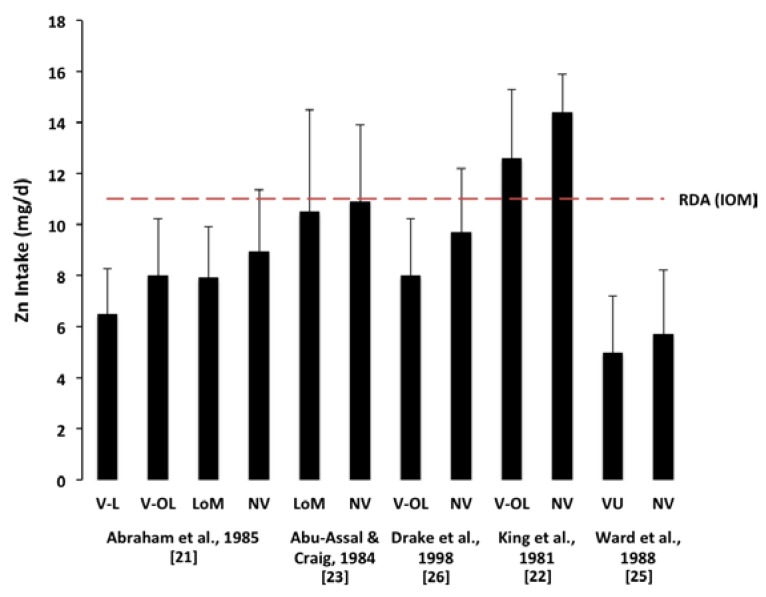
Relationship of dietary zinc intake to Recommended Daily Allowance (RDA). Of the five studies included in the meta-analysis of dietary zinc intake, the participants of one study [22] met the RDA of 11 mg/day established by the Institute of Medicine (IOM) [28]. Results are expressed as mean ± SD. Abbreviations: V-L, lacto-vegetarian; V-OL, ovo-lacto-vegetarian; LoM, low meat; VU, vegetarian undefined; NV, non-vegetarian.

The mean dietary zinc intake in four studies [21,23,25,26] was lower than amounts recommended for pregnancy [28] in both the vegetarian and NV populations (Figure 3). The lowest dietary zinc values were recorded in the study conducted in India [25], with both the vegetarian and NV groups having zinc intakes less than 6 mg/day. Two [23,26] studies reported the amount of zinc ingested from supplements; in the first study [23], in which only NV women were reported to consume zinc supplements, inclusion of supplemental zinc in the measurement of total zinc intake resulted in the NV group meeting the RDA, while in the second study [26] the zinc intake remained lower than the RDA in both the vegetarian and NV groups. One study described but did not quantify the intake of supplemental zinc [22].

### 3.2. Zinc Biomarkers in Pregnancy

Four of the studies [22,23,24,25] that compared dietary zinc intake between pregnant vegetarians and NV controls additionally assessed biomarkers of zinc status. The available data were insufficient to allow meta-analyses to be conducted. Three studies found no differences in serum/plasma zinc [22,23,25], urinary zinc [22], and hair zinc [22] between groups. Of those that considered zinc intake from supplements [22,23], one study [22] reported that use of supplemental zinc did not appear to have any effect on plasma, urinary, or hair zinc levels in the pregnant vegetarian and omnivorous women, while the other did not explore the impact of supplemental zinc on zinc biomarkers. The remaining study [24] that assessed biomarkers of zinc status differed from the others in that it measured concentrations of zinc in serum, urine, and hair at more than one time point. The vegetarian and control groups both demonstrated a pregnancy-associated fall in the plasma zinc concentration and an increase in urinary zinc levels during the study. Although no differences over time were shown between vegetarians and NV in plasma zinc measurements, the urinary zinc concentration was lower in vegetarians than NV controls at all time points.

### 3.3. Functional Outcome in Pregnancy

The most common functional outcomes assessed were period of gestation (3 studies [23,24,26]) and birth weight (5 studies [22,23,24,25,26]). No differences were found between vegetarian populations and their respective control groups in either outcome (Table 3). No studies observed relationships between measures of zinc status and either outcome.

In comparing vegetarian and control groups, two of the studies [24,26] explicitly adjusted for variables that may influence birth weight, such as gestational age, sex of the infant, maternal size, and smoking habit, and one study [25] alluded to an adjustment for maternal weight. Birth weight (crude and adjusted) was similar in infants born to UK immigrant and indigenous Hindu Asian populations [25], regardless of dietary pattern; period of gestation was reported to be shorter and infants lighter in both the vegetarian and NV Asian immigrant populations compared to UK Europeans [24].

**Table 3:** Functional outcomes of pregnancy assessed in included studies ^1^.

Study (author, year)	Vegetarian Type ^2^	Period of Gestation ^3,4^ (week)	Birth Weight ^3,4^ (g)	Other Pregnancy Outcomes
King, Stein & Doyle, 1981 [22]	V-OL	-	3514 ± 573	-
	NV	-	3294 ± 633	
Abu-Assal & Craig, 1984 [23]	LoM	39.5 ± 1.0	3366 ± 375	Apgar score (1 and 5 min)
	NV	40.5 ± 1.0	3634 ± 547	
Campbell-Brown *et al.*, 1985 [24]	VU	38.6 ± 1.5	2905 ± 517	Spontaneous delivery
	NV	38.6 ± 2.2	2926 ± 635	
Ward *et al.*, 1988 [25]	VU	--	2885 ± 547	-
	NV		2904 ± 383	
Drake, Reddy & Davies, 1998 [26]	V-OL	40.3 ± 1.9	3539 ± 590	Mode of deliveryLength of infantHead circumference
	NV	40.3 ± 1.3	3403 ± 392	

^1^ No functional outcomes were assessed in Abraham *et al.*, 1985 [21]; ^2^ Abbreviations: LoM, low meat; NV, non-vegetarian; V-OL, ovo-lacto vegetarian; VU, vegetarian undefined; ^3^ results expressed as mean ± SD; ^4^ no significant differences were reported between vegetarian and NV control groups.

### 3.4. Quality Assessment

Using the ADA Quality Criteria Checklist [27], five [21,22,23,24,25] of the six included studies were assigned a quality rating of neutral. The remaining and most recent study [26] was assigned a quality rating of positive, suggesting a low risk of bias. No studies were excluded from analysis as a result of the quality assessment.

## 4. Discussion

The present meta-analysis of observational studies shows that pregnant vegetarian women have a lower dietary zinc intake than their omnivorous counterparts. In contrast, the balance of evidence evaluated in the present systematic review suggests that there is no difference between groups in biomarkers of zinc status (concentrations of zinc in serum/plasma, urine, hair) or in functional outcomes associated with pregnancy (period of gestation, birth weight).

The current recommended dietary intake for zinc in pregnant women aged 19–50 years is 11 mg/day in Australia/New Zealand [4], Canada [29], and USA [28]. In all but one [22] of the studies included in the present meta-analysis, mean dietary zinc intake was lower than recommended in both the vegetarian and NV populations; the concern that zinc intakes are insufficient to provide the minimum requirement in pregnancy therefore applies to both groups. Vegetarians may be at additional risk, given the present finding that pregnant vegetarians consume 1.4 mg/day less dietary zinc than NV populations, which represents a difference of approximately 13% of the recommended daily allowance (RDA [28]); although the difference is modest, the lower bioavailability of plant-based zinc sources [4,5] suggests that it may make an important contribution to maternal health.

In order to conclude that dietary zinc intakes are insufficient to meet the additional zinc demands of pregnancy, however, evidence of adverse effects either to the mother or the foetus needs to be established. None of the reports included in this review demonstrated adverse effects of a low zinc diet in either the vegetarian or NV populations. No significant differences were found between groups in period of gestation or birth weight, and dietary zinc intake and serum/plasma zinc, as measures of zinc status, were not related to either outcome. Three [24,25,26] of the included studies stated that they were designed specifically to investigate the relationship between zinc status and functional pregnancy outcomes. Two of these studies [24,25], from the same research group, investigated the relationship between zinc status and pregnancy outcome in pregnant Asian populations; it was observed that birth weights were similar between the infants of UK immigrant and Indian (Gujerati) women, regardless of dietary pattern and notwithstanding a zinc intake in the Gujerati women that was almost half that of the immigrant group [25].

At least three explanations could account for the absence of evidence of adverse maternal or foetal health effects despite reported dietary zinc intakes in pregnancy being at levels below the RDA. In the last decade, few, if any, studies have been conducted to explore the relationship between zinc status and pregnancy outcome in humans, and none that include the effect of vegetarian dietary patterns, hence the relationship between zinc status and pregnancy outcome remains ill-defined [8]. Secondly, dietary zinc intakes may in fact have met the RDA for pregnancy but were under-reported. Of the five studies that qualified for the meta-analysis, two [21,25] assessed dietary zinc intake using a 7-day dietary recall method, while the remaining three [22,23,26] employed a 3-day estimated diet record. Although its use is not always practicable, the most accurate method for the measurement of zinc intake is the weighed dietary method [30]. Further, whether the included studies accounted for supplemental zinc use in the measurement of dietary zinc intake is unclear in some instances. The third explanation for the absence of adverse consequences is that physiologic adaptations in both the vegetarian and omnivorous populations allowed zinc status to be maintained despite low dietary zinc intakes. It has been reported that homeostatic mechanisms allow healthy adults to adapt to low zinc diets, including those that are plant-based [7]. It is plausible that such adaptations in zinc metabolism are sufficient to maintain zinc status also during periods of increased requirement, such as pregnancy [31]. If correct, this rationalisation implies that the RDA for zinc in pregnancy requires further examination and review. The RDA is continually under appraisal; the current recommendation of 11 mg/day is lower than the amount (20 mg/day [32]) applied in the five studies conducted in the 1980s [21,22,23,24,25] and higher than the amount (7 mg/day [33]) employed in the single study published in the following decade [26]. RDAs are estimates with a considerable degree of uncertainty, particularly where scientific evidence is limited. Until the relationship between dietary zinc intake, zinc status, and pregnancy outcome is understood more completely, a cautious interpretation of the amount of dietary zinc required in pregnancy is merited.

The assessment of zinc status in pregnancy is complicated by a number of physiologic adjustments in zinc metabolism that occur during gestation, including a decline in the plasma zinc concentration [34] and an increase in the concentration of urinary zinc [35]. In the present systematic review, no differences were found between vegetarian and NV pregnant women in third trimester concentrations of serum/plasma, urinary, or hair zinc, with one exception. One study [24] investigated the zinc status of vegetarian pregnant women compared to controls at multiple time points; although no differences were found between vegetarians and NV in plasma zinc measurements, the urinary zinc concentration was lower in vegetarians than NV at all time points, which warrants further investigation.

A key limitation of the existing literature on vegetarian nutrition relates to the lack of consistency in definitions ascribed to vegetarian populations for research purposes [6]. In strict terms, an individual is considered a vegetarian (lacto-, ovo-, or lacto-ovo) if they abstain from eating all flesh foods (meat, poultry, fish, shellfish). In categorising populations for the meta-analysis, it was observed in two studies [21,23] that participants were described as vegetarian despite consuming limited amounts of animal flesh. The meta-analysis therefore was re-analysed after exclusion of the two LoM comparisons in order to investigate the effect on zinc intake of diets that adhere to strict interpretations of vegetarian dietary patterns. Applying this threshold, pregnant vegetarians were found to consume 1.5 mg/day less dietary zinc than NV, representing a difference of approximately 14% of the RDA. The slightly greater difference in zinc intake after removal of LoM comparisons supports the suggestion in the largest included study [21] that the amount of zinc ingested reflects differences in total animal protein intake among the different dietary patterns. Clarity and specificity in defining study populations are necessary if research in this field is to contribute effectively to the determination of zinc intake requirements in vegetarians.

Strengths of the current review include the use of the random effects model of meta-analysis, which allows for heterogeneity among studies. Overall, the systematic review highlights the limited availability of research comparing the zinc intake and status of pregnant vegetarian and NV populations. Limitations arising from the restricted availability of published information and the small sample size of many of the selected studies include the inability to perform meta-analyses of biomarkers of zinc status and functional outcomes; similarly, secondary analyses of dietary zinc intake were unable to be undertaken to investigate the impact, for example, of vegetarian type, country classification (low-, middle-, high-income [6]), stage of pregnancy when dietary zinc was measured, supplement use, and dietary methodology employed.

The present systematic review underscores the need for updated investigations in the field of vegetarian research, both as it relates to pregnancy and more broadly to zinc status throughout the life cycle. Of the 6 studies that qualified for inclusion in the review, only one was assessed as being of ‘positive’ quality, with the quality of the remaining 5 being categorised as ‘neutral’. The quality ratings appear to relate to the date of study authorship as much as intrinsic quality concerns; for example, only the more recent of the included papers discussed study limitations, which likely reflects the increase over time in emphasis on appropriate reporting standards and the availability of reporting guidelines for studies. More generally, the designs of future studies seeking to assess the zinc status of vegetarians compared to the general population need to use precision when taking into account differences in vegetarian populations [36]. In addition, updated information on the sources and bioavailability of zinc from vegetarian and omnivorous diets is required, in both high- and lower-income countries. An increase in the availability and/or use of supplements and fortified foods is likely to have affected positively the zinc status of some groups. Further, modern methods of food processing may have altered the dietary content of phytic acid [PA], a potent inhibitor of zinc absorption, suggesting the need also for revised phytate data that is location-specific and reported as an index of zinc bioavailability. Methodologies for laboratory assessments need to be appropriate and consistent; for instance, the International Zinc Consultative Group protocols [37] are recommended for the measurement of serum/plasma zinc, and methods of PA analysis need to quantify individual inositol phosphates (IP) as only IP5 and IP6 appear to affect zinc absorption [38].

## 5. Conclusions

It is now recognised that appropriately planned vegetarian diets are nutritionally adequate and suitable for all stages of the life cycle [39]. In practice, the findings of the present meta-analysis suggest that pregnant vegetarian women have lower dietary zinc intakes than NV control populations. The effects of the disparity in zinc intake between pregnant vegetarians and omnivores are unclear, with the evidence to date showing no difference between groups in serum/plasma zinc concentration or functional pregnancy outcomes. Further information is needed to determine whether physiological adaptations in zinc metabolism are sufficient to meet maternal and foetal requirements during pregnancy across a range of zinc intakes and dietary patterns. In the meantime, strategies known to increase the zinc content and bioavailability of vegetarian diets should continue to be recommended prior to conception and throughout pregnancy, including as appropriate the use of food preparation and processing methods that inactivate PA, the consumption of foods fortified with zinc, and low dose zinc supplementation [40]; pregnant omnivorous women also are likely to benefit from advice that increases their zinc intake to recommended daily levels.
